# Height and weight predict cross‐sectional area of the peroneus brevis and longus tendons: Magnetic resonance imaging‐based analysis of 164 adults

**DOI:** 10.1002/jeo2.70492

**Published:** 2025-11-03

**Authors:** Rafał Zych, Dan Mocanu, Katarzyna Bokwa‐Dąbrowska, Dawid Dziedzic, Pawel Szaro

**Affiliations:** ^1^ Department of Clinical and Descriptive Anatomy Medical University of Warsaw Warsaw Poland; ^2^ Department of Musculoskeletal Radiology Sahlgrenska University Hospital Gothenburg Sweden; ^3^ Department of Radiology, Institute of Clinical Sciences, Sahlgrenska Academy University of Gothenburg Gothenburg Sweden

**Keywords:** autograft planning, MRI‐based tendon sizing, peroneus brevis, peroneus longus, predictors of tendon size

## Abstract

**Purpose:**

To determine whether height, weight and body mass index (BMI) are associated with the cross‐sectional area of the peroneus brevis and longus tendons on magnetic resonance imaging (MRI). Understanding these associations may assist in autograft selection and evaluation of residual tendon function after graft harvest.

**Methods:**

This retrospective study included 164 adult patients (mean age 41.6 ± 15.7 years; 52% female) who underwent 3T ankle MRI between 2018 and 2024. Patients with peroneal tendon pathology (e.g., split tears, tendinosis), prior surgery or artefacts (e.g., motion or metal‐related signal loss) were excluded. Cross‐sectional areas were measured 1 cm above the lateral malleolus on axial proton density‐weighted images. Height and weight were obtained from pre‐MRI exam documentation. Associations with tendon cross‐sectional area were evaluated using multivariable linear regression models adjusted for age and side.

**Results:**

Mean cross‐sectional areas were 14.0 ± 4.2 mm² for the peroneus brevis and 19.3 ± 4.7 mm² for the peroneus longus. Peroneus brevis area was significantly associated with height (*β* = 12.9 mm²/m, *p* < 0.001) and age (*β* = 0.06 mm²/year, *p* < 0.001). Peroneus longus area was associated with weight (*β* = 0.07 mm²/kg, *p* = 0.01). No significant associations were found with BMI. All findings were robust in sensitivity analyses.

**Conclusions:**

Height and weight are reliable predictors of peroneal tendon size; BMI is not. MRI is a standardised, noninvasive tool for individualised graft planning and assessment of postoperative tendon integrity. These findings may assist in autograft planning and in evaluating residual tendon function after graft harvest.

**Level of Evidence:**

Level III.

## Graphical abstract

Weight predicts peroneus longus size, height and age predict peroneus brevis size, and body mass index shows no significant association—based on magnetic resonance imaging analysis of 164 healthy adults. Scatterplots showing the relationship between tendon cross‐sectional area and body mass index, weight and height. Left panels: peroneus brevis; right: peroneus longus. Each point represents one individual; dashed lines show linear regression trends.

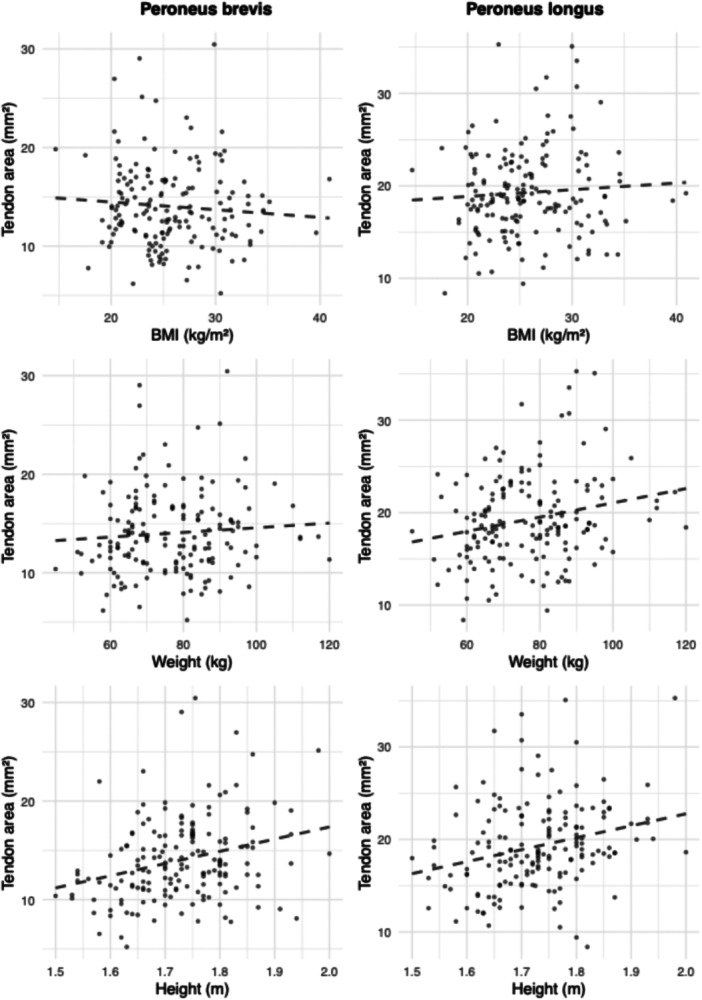

**Abbreviations**
ACL: anterior cruciate ligament
BMI: body mass index
CI: confidence interval
CSA: cross‐sectional area
ICC: intraclass correlation coefficient
LOESS: locally estimated scatterplot smoothing
MRI: magnetic resonance imaging
PCL: posterior cruciate ligament
SD: standard deviation

## INTRODUCTION

The peroneal tendons stabilise the ankle and ensure effective gait [6, 24]. The peroneus brevis inserts on the base of the fifth metatarsal and plays an essential role during the stance phase of gait, and the peroneus longus passes beneath the foot to insert on the first metatarsal and medial cuneiform, contributing to first ray plantarflexion and stabilisation of the transverse arch [10, 16]. The peroneus longus tendon is now used as an autograft in anterior cruciate ligament (ACL) and posterior cruciate ligament (PCL) reconstructions [9]. Compared with traditional hamstring grafts, peroneus longus autografts offer several advantages, including consistent graft diameter and reduced donor‐site morbidity, such as less postoperative pain and preserved knee flexor strength [1]. Clinical outcomes following graft harvest have been favourable, with most patients demonstrating good ankle function [5, 18]. While minor reductions in eversion strength have been reported, these are typically not functionally limiting [20, 21]. Harvesting half of the peroneus longus tendon allows residual function to be maintained [12, 25]. This is particularly important because, after graft harvest, the peroneus brevis remains the only tendon within the retromalleolar groove and must compensate for the loss of peroneus longus function, leading to increased mechanical load during ankle stabilisation and eversion.

Knowing the graft size before ACL reconstruction is crucial for a successful outcome. Smaller grafts, especially those 7 mm or less, have been linked to higher failure and revision rates, as well as poorer knee function [8]. Given its widespread clinical use and previous studies reporting conflicting results, we included body mass index (BMI) alongside height and weight to evaluate its predictive value for tendon size. Rhatomy et al. [19] studied 39 patients undergoing ACL reconstruction and found a weak but statistically significant correlation between BMI and intraoperative peroneus longus diameter. Wierer et al. [25] evaluated 64 patients who underwent anterior half peroneus longus tendon harvest and found no correlation between BMI and tendon size in multivariate analysis, while height, rather than BMI, was the strongest predictor of graft size. A larger retrospective study by Song et al. [22], involving 156 patients, also found no significant association between BMI and graft diameter. Compared to surgical measurements, which can vary with technique, lack interrater reliability and provide limited anatomical precision, magnetic resonance imaging (MRI) is a standardised, noninvasive and anatomically precise method to assess tendon morphology [4, 15, 27, 28]. Thus, MRI permits standardised measurement of tendon cross‐sectional area, independent of surgical variability and facilitates larger sample sizes. A previous MRI‐based study by Albano et al. [2], involving 113 patients, reported a weak correlation between weight and peroneus longus diameter and found no significant associations with height or BMI. Yet, no previous study has assessed the morphology of the peroneus brevis tendon, which may be clinically relevant for long‐term follow‐up after peroneus longus harvest. In a large ultrasound study of 199 patients, Jackson et al. reported normative thickness values for the peroneus longus and brevis tendons but did not investigate their relationship to anthropometric measures such as height, weight or BMI [11].

Overall, there is a lack of studies based on larger populations using noninvasive methods with interrater reliability to examine the relationship between BMI, weight and height with the cross‐sectional area of the peroneus longus tendon, and existing research has yielded inconsistent results. Additionally, no studies have investigated this relationship in the peroneus brevis tendon. This gap is particularly important given the clinical relevance of tendon size in graft harvesting for ACL/PCL reconstructions. By using MRI, this study provides precise, noninvasive measurements of peroneal tendon morphology, offering an advantage over surgical findings, which can lack anatomical accuracy and be influenced by surgical variability.

This study aims to investigate whether height, weight and BMI are associated with the cross‐sectional area of the normal peroneus longus and peroneus brevis tendons on MRI. We hypothesised that height and weight would be positively associated with the cross‐sectional area of the peroneus tendons, while BMI would show weaker or no association, based on inconsistent findings in previous literature.

## METHODS

### Study design

We conducted a retrospective cross‐sectional study and reported it in accordance with the STROBE guidelines for observational research [23].

### Setting

Eligible examinations were identified from the institutional radiology archive. All data were acquired between 1 February 2018, and 31 December 2024, as part of routine clinical ankle MRI protocols. The analyses were conducted between January and April 2025.

### Participants

Eligible participants included adults (≥18 years) who underwent ankle MRI and had normal peroneal tendon morphology on proton density and T2‐weighted images. Cases with split tears of the peroneus brevis and longus, fractures, infections, ganglion cysts or tumours, transverse ruptures of the peroneus brevis and longus, postoperative changes, the presence of metal hardware, and MRI‐related artefacts were excluded. Additional exclusions were made for patients under 18 years of age, those who had missing weight and height data, and for repeated MRI examinations of the same individual.

**Figure 1:**
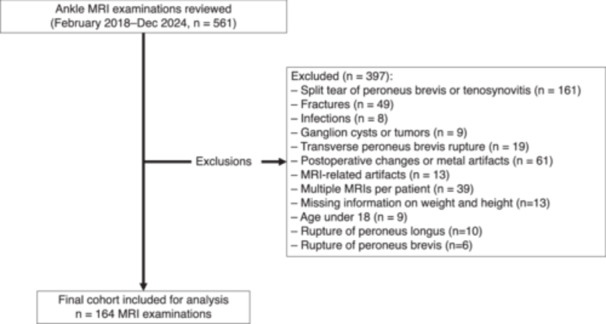
Flow chart of magnetic resonance imaging (MRI) examinations included in the study.

A total of 164 MRI examinations of healthy adult were included in the final analysis (Figure 1). Among these, 86 MRI examinations were from females (45 left, 41 right) and 78 from males (40 left, 38 right). The mean age was 41.6 years (SD 15.7), with a BMI range of 14.7−40.9 kg/m². The mean body weight was 76.8 kg (SD 13.9), and the mean height was 1.73 m (SD 0.10).

### Variables

The primary outcome variables were the cross‐sectional areas (in mm²) of the peroneus brevis and longus tendons. Measurements were taken on transverse sections on proton density‐weighted images at the level of the lateral malleolus, 1 cm above the apex of the lateral malleolus.

Predictor variables included: (a) BMI, calculated as weight (kg) divided by height squared (m²), (b) weight (kg) and (c) height (m) as continuous variables, (d) age (years) and (e) side (left or right). BMI was calculated from patient‐reported height and weight collected prior to MRI examination. Self‐reported height and weight show high agreement with measured values and are appropriate for BMI estimation [7, 14]. No additional confounders or effect modifiers were prespecified, but side‐stratified analyses were performed secondarily.

### Data sources and measurement

MRI data were acquired on 3T systems using standard ankle imaging protocols. Based on findings from a preliminary study, the cross‐sectional shape of both the peroneus brevis and longus tendons is maintained at the level of the lateral malleolus, supporting the use of a single representative slice for the analyses. Measurements were performed on axial proton density‐weighted images approximately 1 cm above the apex of the fibula, corresponding to the first slice proximal to the tip of the lateral malleolus. Tendon outlines were manually segmented using the freehand tool in ImageJ (Files S1 and S2), and cross‐sectional area (in mm²) was automatically calculated. The macro used for segmentation and area extraction had been previously validated in the same imaging context (File S2) [26]. All segmentations were performed independently by one musculoskeletal radiologist (P.S.) and one trained researcher (R.Z.), both blinded to patient identity and anthropometric data (weight, height and BMI). We assessed interrater agreement using the intraclass correlation coefficient [ICC(A, 1)], based on a two‐way random effects model with absolute agreement and single measures. ICCs were calculated separately for the peroneus longus and peroneus brevis cross‐sectional area measurements. Interpretation of ICC values followed the guidelines proposed by Koo and Li: [13] values <0.5 indicate poor reliability, 0.5–0.75 moderate reliability, 0.75–0.9 good reliability and >0.9 excellent reliability. The final value was defined as the mean of the two raters' measurements.

Anthropometric data, including self‐reported height and weight, were extracted from the radiology information system. Previous studies have demonstrated high agreement between self‐reported and measured BMI [7, 14]. All images were inspected for quality control, and cases with significant artifacts were excluded by a senior musculoskeletal radiologist (P.S.).

### Bias

As a retrospective single‐centre study relying on self‐reported anthropometric data, the study may be subject to selection bias due to its clinical setting, and reporting bias related to possible inaccuracies in patient‐reported height and weight.

### Quantitative variables

Anthropometric variables (BMI, weight and height) and age were analysed both as continuous variables and grouped into quartiles for secondary descriptive visualisation. Tendon areas (mm^2^) were used in their raw continuous form in all regression models.

### Statistical methods

#### Study size

Sample size was determined a priori using G*Power 3.1 (Heinrich‐Heine‐Universität Düsseldorf, Germany) for a multiple linear regression model (fixed model, R² deviation from zero) with four predictors. Effect size *f*2 = 0.081 *f*2 = 0.081 was estimated from a preliminary analysis of 50 patients. To achieve 80% power (1−β = 0.80) at a significance level of 0.05, the required total sample size was calculated to be 153 participants. A total of 164 patients were included to account for potential exclusions and to enable subgroup and sensitivity analyses.

#### Main analyses

Associations between anthropometric variables and tendon cross‐sectional areas were assessed using separate multiple linear regression models for each tendon. Each model included either BMI, weight, or height as the primary exposure and was adjusted for age and side. Prior to model selection, we evaluated the functional form of each continuous predictor using locally estimated scatterplot smoothing (LOESS) plots to screen for potential nonlinear trends. These visualisations supported the use of linear modelling, as the relationships between tendon size and height or weight appeared approximately linear. BMI demonstrated minor curvature in some plots but showed weak and inconsistent associations overall. For clarity and interpretability, we proceeded with linear models. All regression estimates include 95% confidence intervals. Unstandardised beta coefficients (β), 95% confidence intervals (CIs) and *p*‐values are reported. Although splines could capture potential nonlinear effects, we favoured linear models for simplicity and interpretability, as exploratory LOESS plots did not reveal consistent curvature across predictors.

#### Subgroup and interaction analyses

To examine potential lateral effects, stratified regressions were performed by side (left/right).

#### Missing data

No missing data were identified. All included cases had complete anthropometric and imaging data.

#### Sensitivity analyses

Model robustness was evaluated using two approaches: (e1) Robust linear regression (M‐estimation with Huber weighting) to reduce sensitivity to outliers; (e2) Refitting models after excluding observations with Cook's distance exceeding the 4/n threshold. Diagnostic plots, including residual‐vs‐fitted, Q–Q plots, and Cook's distance, are provided in Supporting Information S3: Figure A1. Collinearity was assessed using generalised variance inflation factors, with values > 2 flagged.

All analyses were performed in R (version 4.4.3).

## RESULTS

### Participants

**Figure 2:**
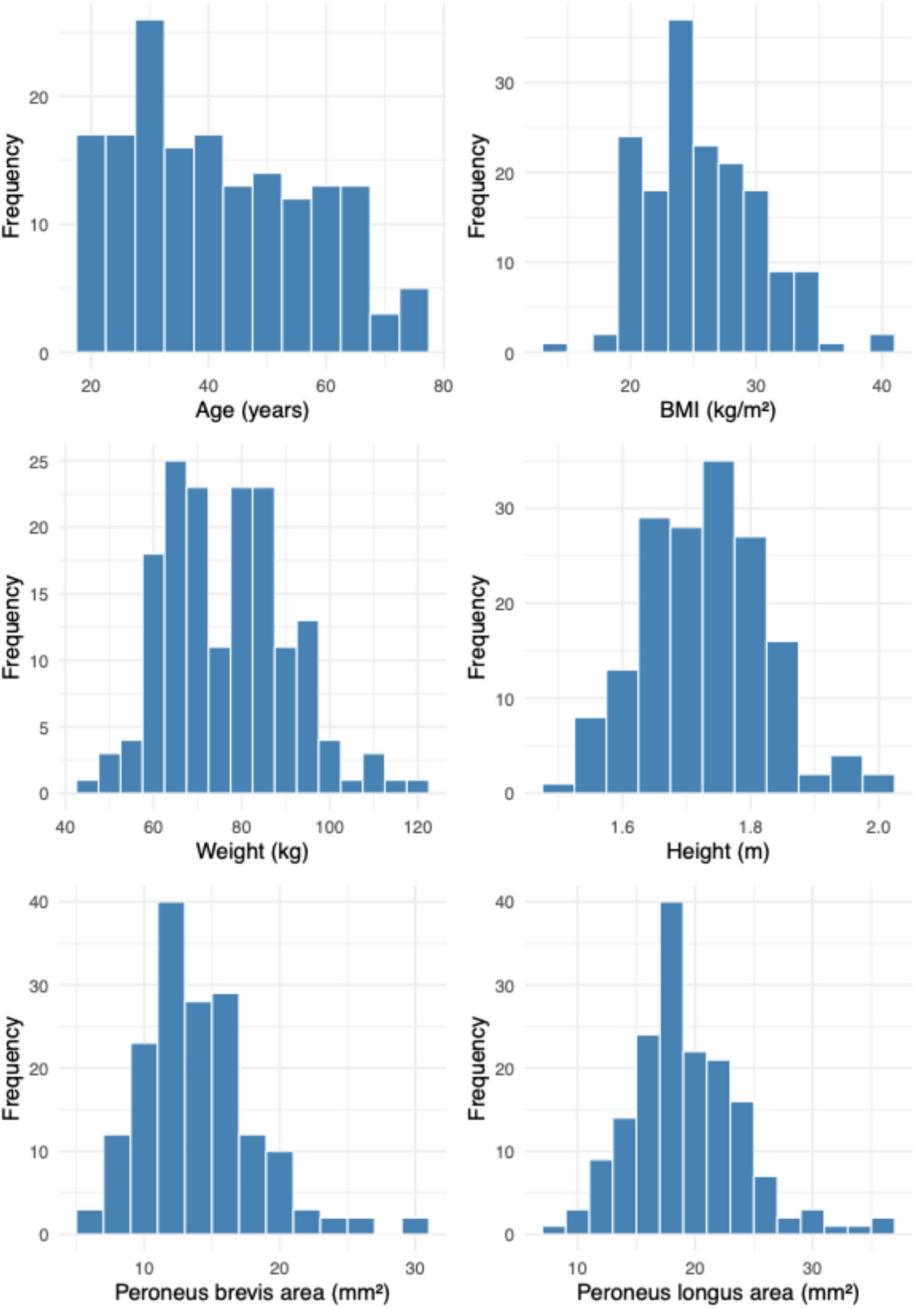
Distribution of continuous variables in the study cohort, including age, BMI, weight, height and cross‐sectional areas of the peroneus brevis and longus tendons.

Mean cross‐sectional areas were 14.0 mm² (SD 4.2) for the peroneus brevis and 19.3 mm² (SD 4.7) for the peroneus longus (Table 1, Figure 2).

**Table 1:** Demographic and anatomical characteristics of the study cohort.

Variable	Mean	SD	Min	Q1	Median	Q3	Max
Age (year)	41.61	15.65	18	28	40	54	77
BMI (kg/m²)	25.76	4.45	14.68	22.39	25.03	28.4	40.9
Weight (kg)	76.82	13.93	45	66	77	86	120
Height (m)	1.73	0.1	1.5	1.66	1.73	1.8	2
Peroneus brevis cross sectional area (mm^2^)	14.02	4.22	5.21	11.18	13.46	16.5	30.45
Peroneus longus cross sectional area (mm^2^)	19.26	4.74	8.38	16.3	18.59	22.15	35.29

*Note*: Continuous variables are presented as mean (±SD), and categorical variables as counts.

Abbreviations: BMI, body mass index; CSA, cross‐sectional area; SD, standard deviation.

### Tendon area distribution

**Figure 3:**
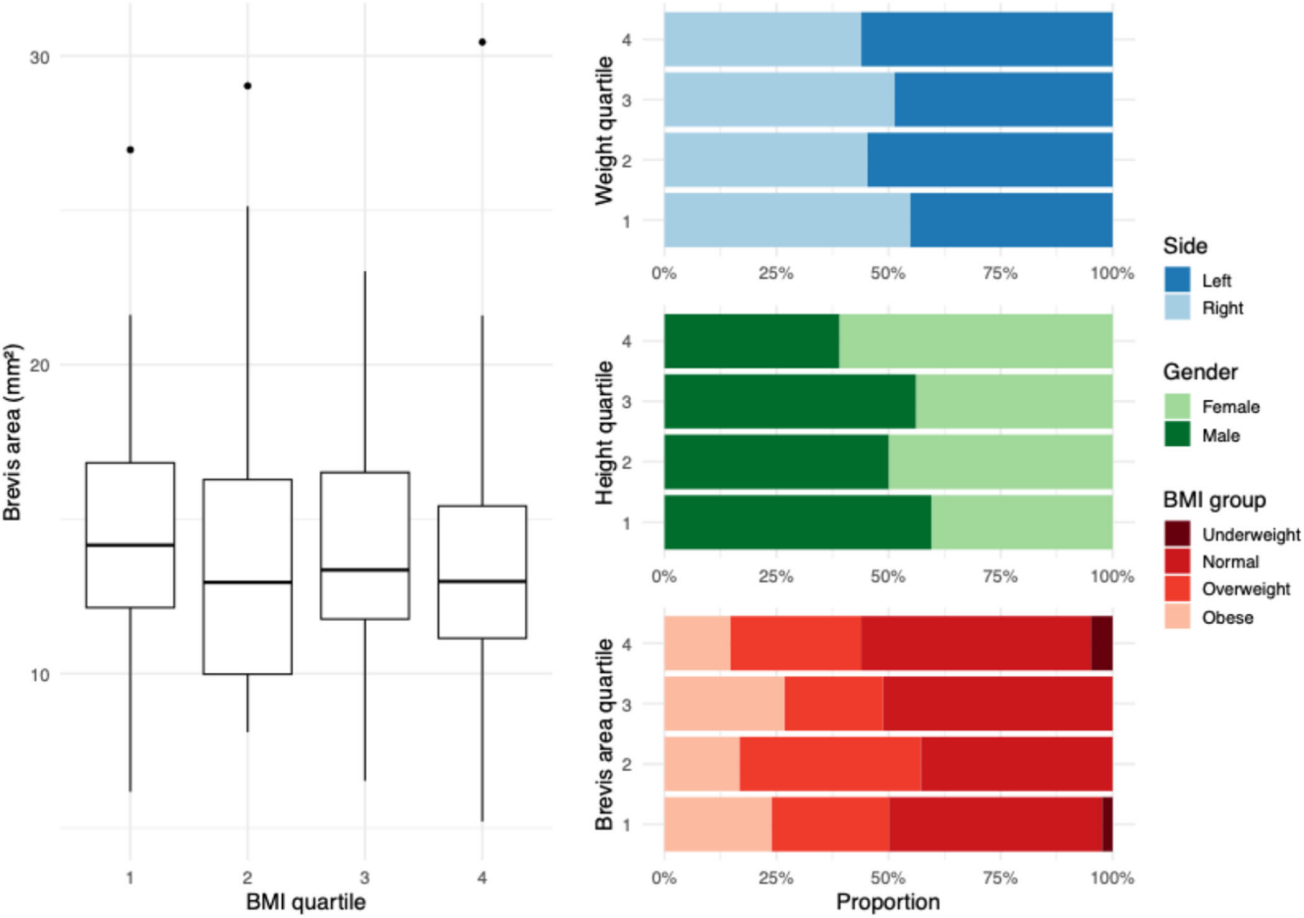
Relationship between anthropometric traits and peroneus brevis cross‐sectional area. Body mass index (BMI) is categorised using established clinical thresholds: underweight (<18.5 kg/m²), normal weight (18.5–24.9), overweight (25–29.9), obese (30–34.9) and extremely obese (≥35). Boxplot of peroneus brevis tendon area across BMI quartiles. Proportion of left and right sides within weight quartiles. Gender distribution across height quartiles. Proportion of BMI categories (underweight, normal, overweight, obese) within peroneus brevis tendon area quartiles. All bar plots are shown as horizontal stacked bars with relative proportions (0%–100%). Quartiles were calculated across the full study cohort.

**Figure 4:**
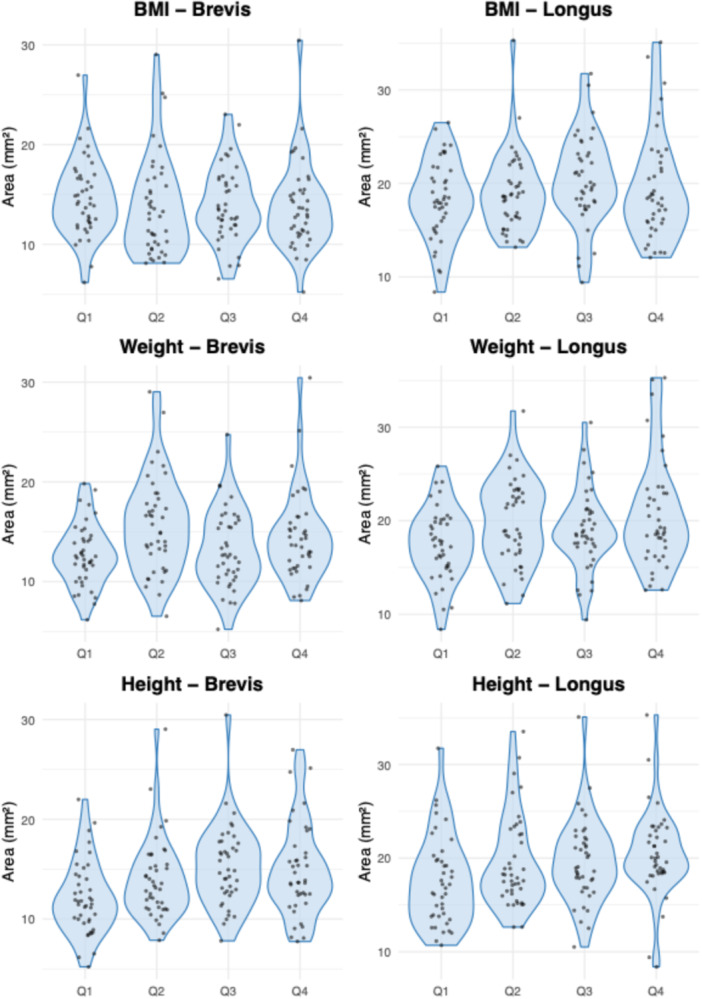
Violin plots of peroneus brevis (marked as Brevis) and peroneus longus (marked as Longus) cross‐sectional area across body mass index (BMI), weight and height quartiles. Violin plots and overlaid jitter points show the distribution of peroneus brevis (left column) and peroneus longus (right column) cross‐sectional areas across quartiles of BMI (top row), weight (middle) and height (bottom). Each point represents an individual.

**Figure 5:**
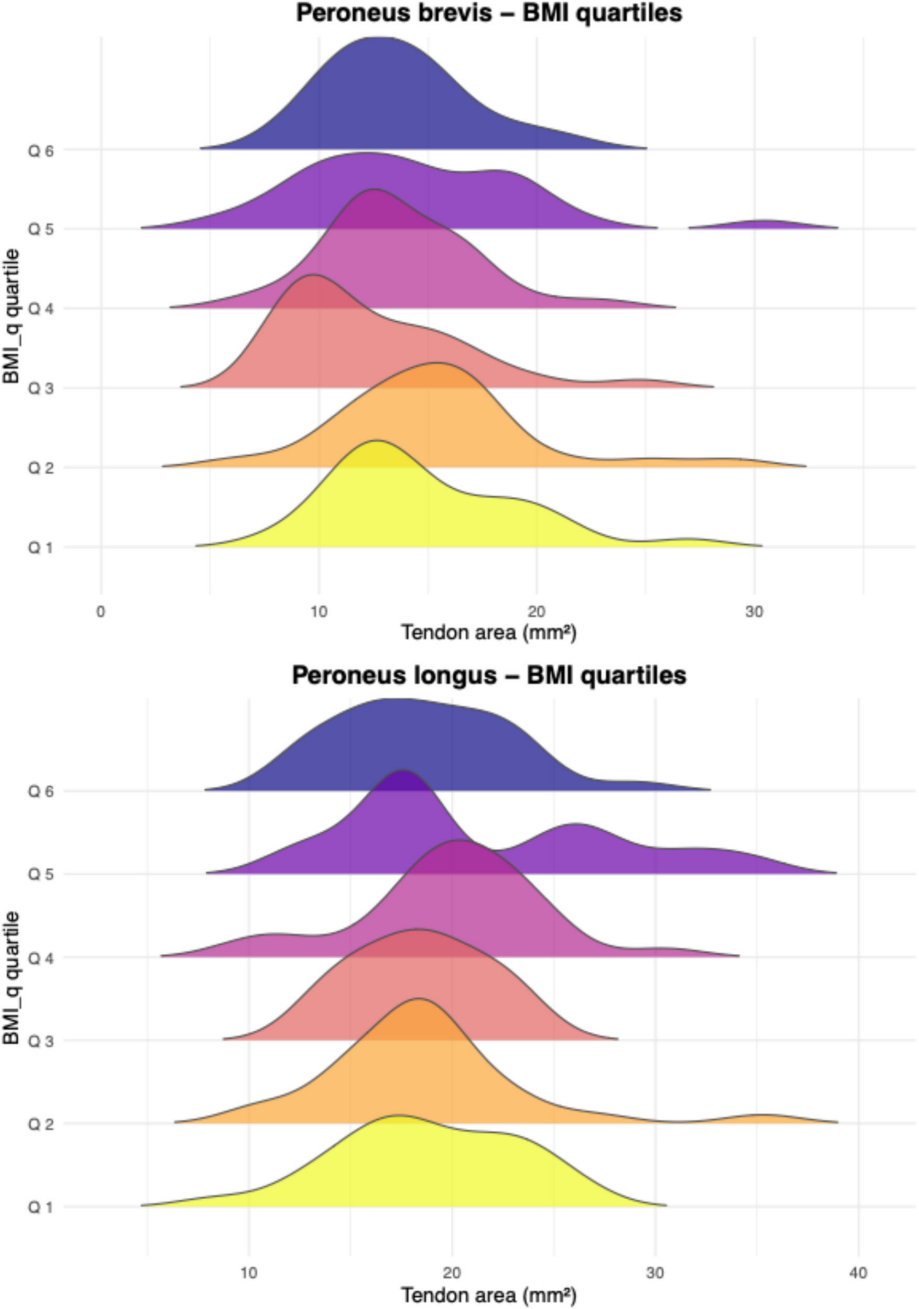
Ridgeline plots of tendon area across body mass index (BMI) quartiles. Each stacked density curve represents the distribution of tendon area for a BMI quartile. Top: peroneus brevis; bottom: peroneus longus. Ridgeline thickness and overlap illustrate the distribution shape and variation across groups.

Interrater agreement was excellent for the cross‐sectional area measurements of both tendons [13]. For the peroneus longus, the ICC(A, 1) was 0.901 (95% CI: 0.861–0.929; *p* < 0.001). For the peroneus brevis, the ICC was 0.915 (95% CI: 0.851–0.947; *p* < 0.001). Tendon cross‐sectional areas appeared to increase across quartiles of BMI, weight and height (Figures 3, 4, 5). Individuals with higher anthropometric values demonstrated broader distributions and higher density peaks. Stratified visualisations suggested subtle asymmetries by sex and side, particularly in weight and height quartiles (Figure 3). Tendon size increased progressively across weight and height quartiles, with the peroneus brevis showing stronger dependence on height and the peroneus longus more influenced by weight.

### Main regression models

**Figure 6:**
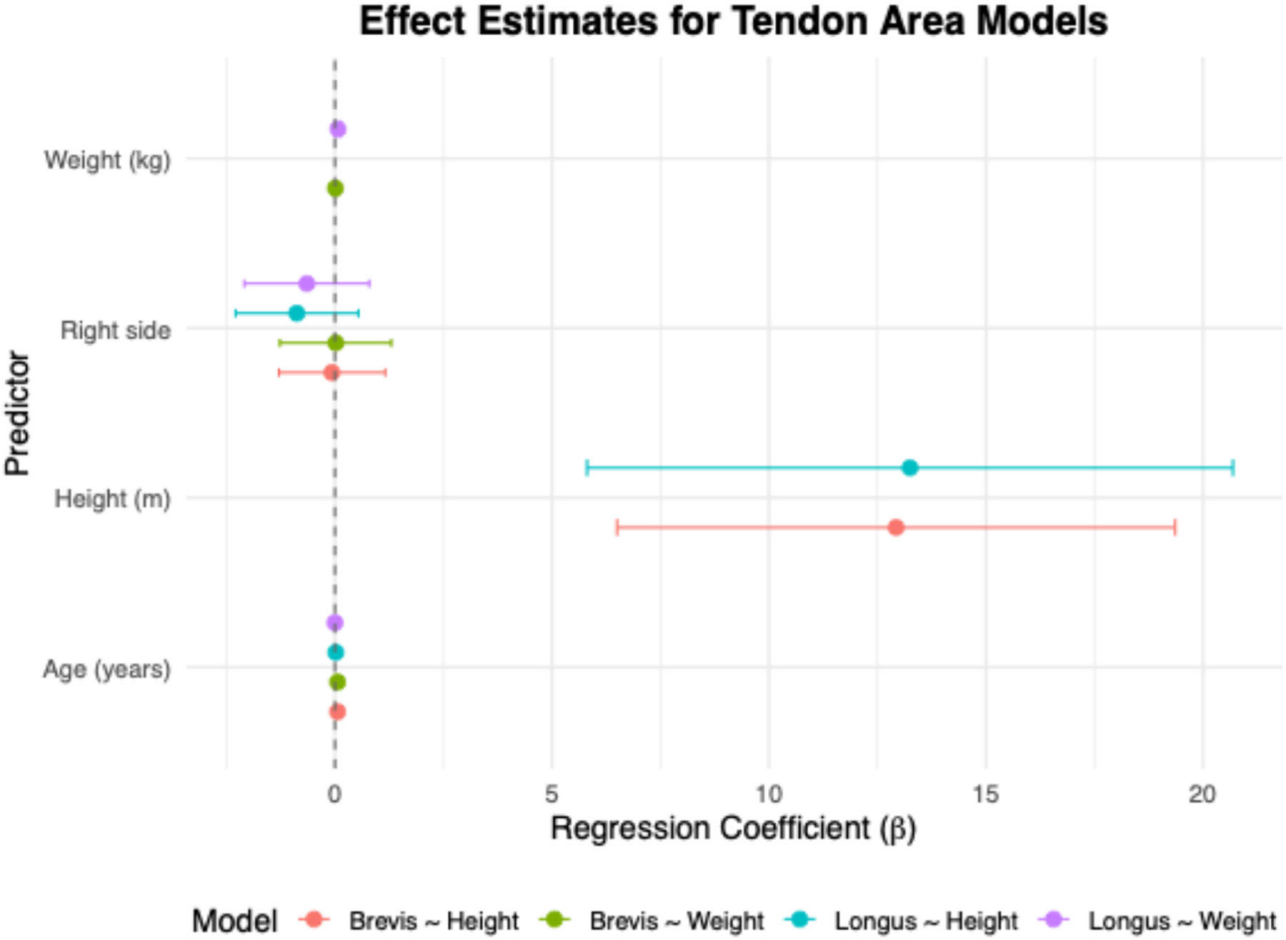
Effect estimates from individual regression models with 95% confidence intervals.

**Figure 7:**
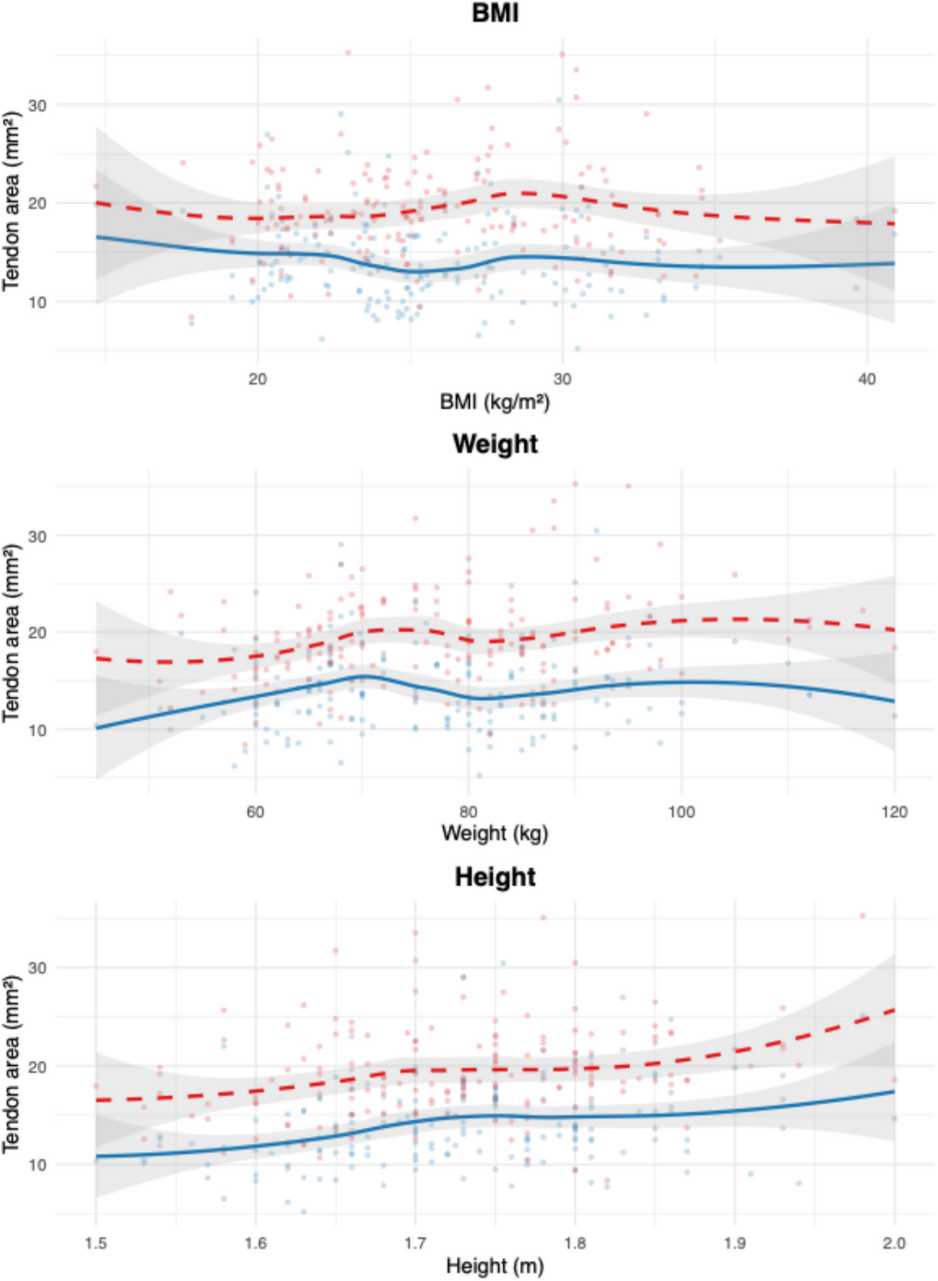
Locally estimated scatterplot smoothing (LOESS)‐plots of tendon area by body mass index (BMI), weight and height. LOESS‐relationships between anthropometric variables and the cross‐sectional areas of the peroneus brevis (solid blue line) and peroneus longus (dashed red line) tendons. Each panel represents a different predictor: BMI (top), weight (middle) and height (bottom). Shaded areas represent 95% confidence intervals. Each point represents one individual observation. A common legend is displayed above.

**Figure 8:**
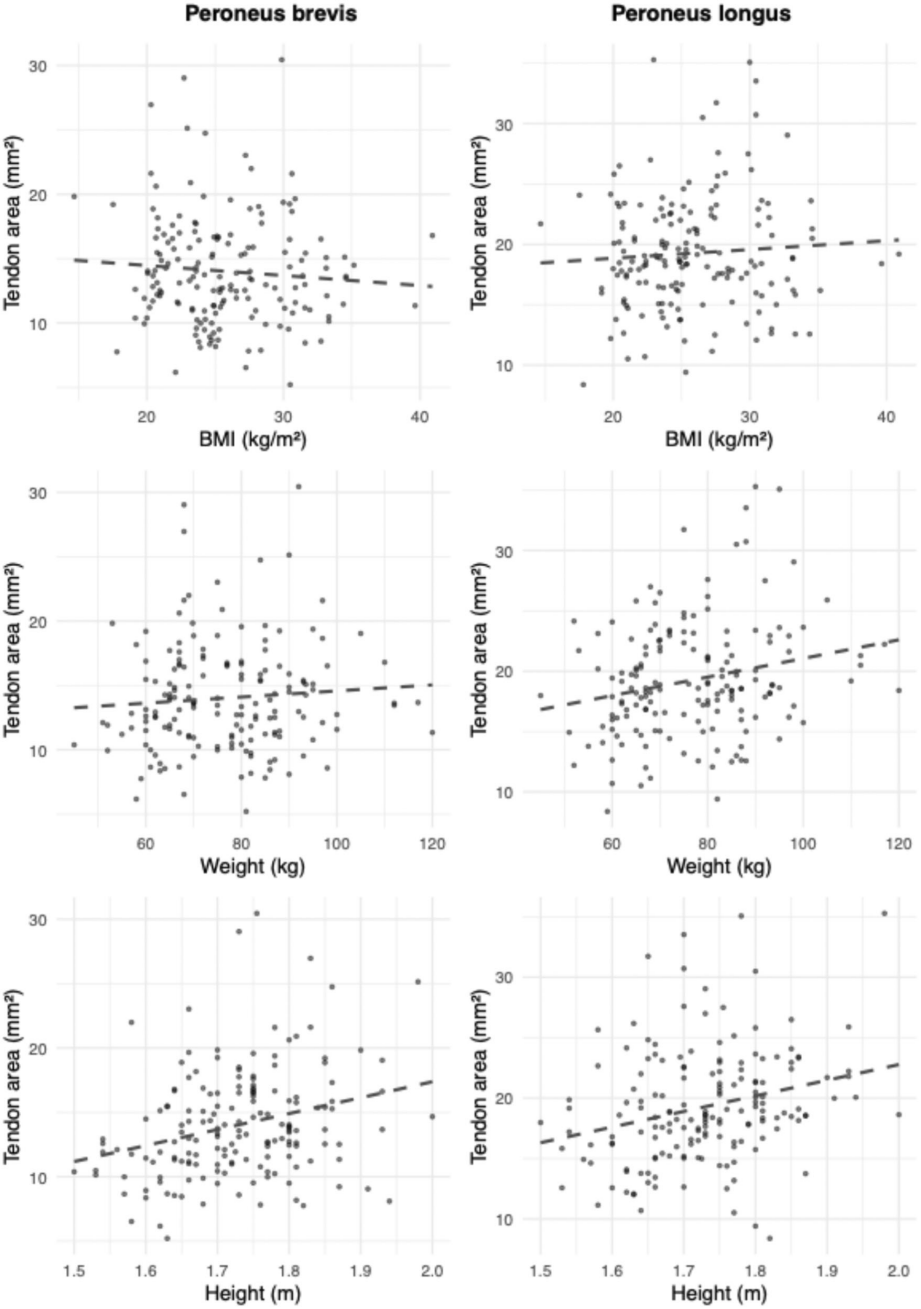
Scatterplots of peroneus tendon area in relation to anthropometric variables. Each panel shows the relationship between tendon cross‐sectional area and an anthropometric measure. The left column displays data for the peroneus brevis tendon, and the right column for the peroneus longus tendon. The top row corresponds to body mass index (BMI), the middle to weight and the bottom to height. Each point represents an individual. Dashed lines indicate linear regression fits to illustrate overall trends.

**Figure 9:**
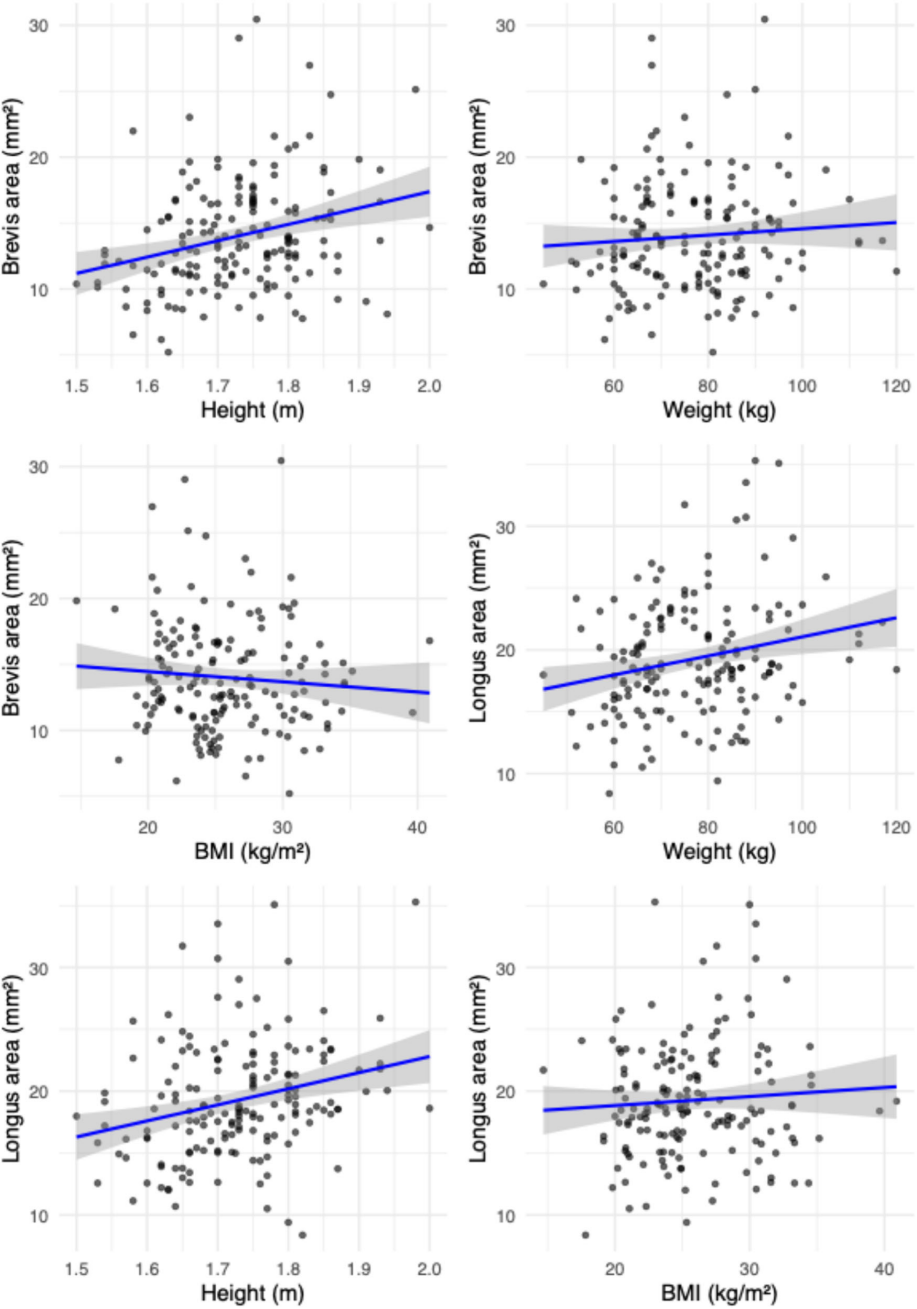
Association between anthropometric variables and peroneal tendon cross‐sectional area. Each panel presents a linear regression with the 95% confidence interval between tendon area (*y*‐axis) and height, weight, or body mass index (BMI) (*x*‐axis). The peroneus brevis tendon is shown in the top two rows, and the peroneus longus tendon in the bottom row. All regressions are unstratified, reflecting overall group‐level associations.

**Figure 10:**
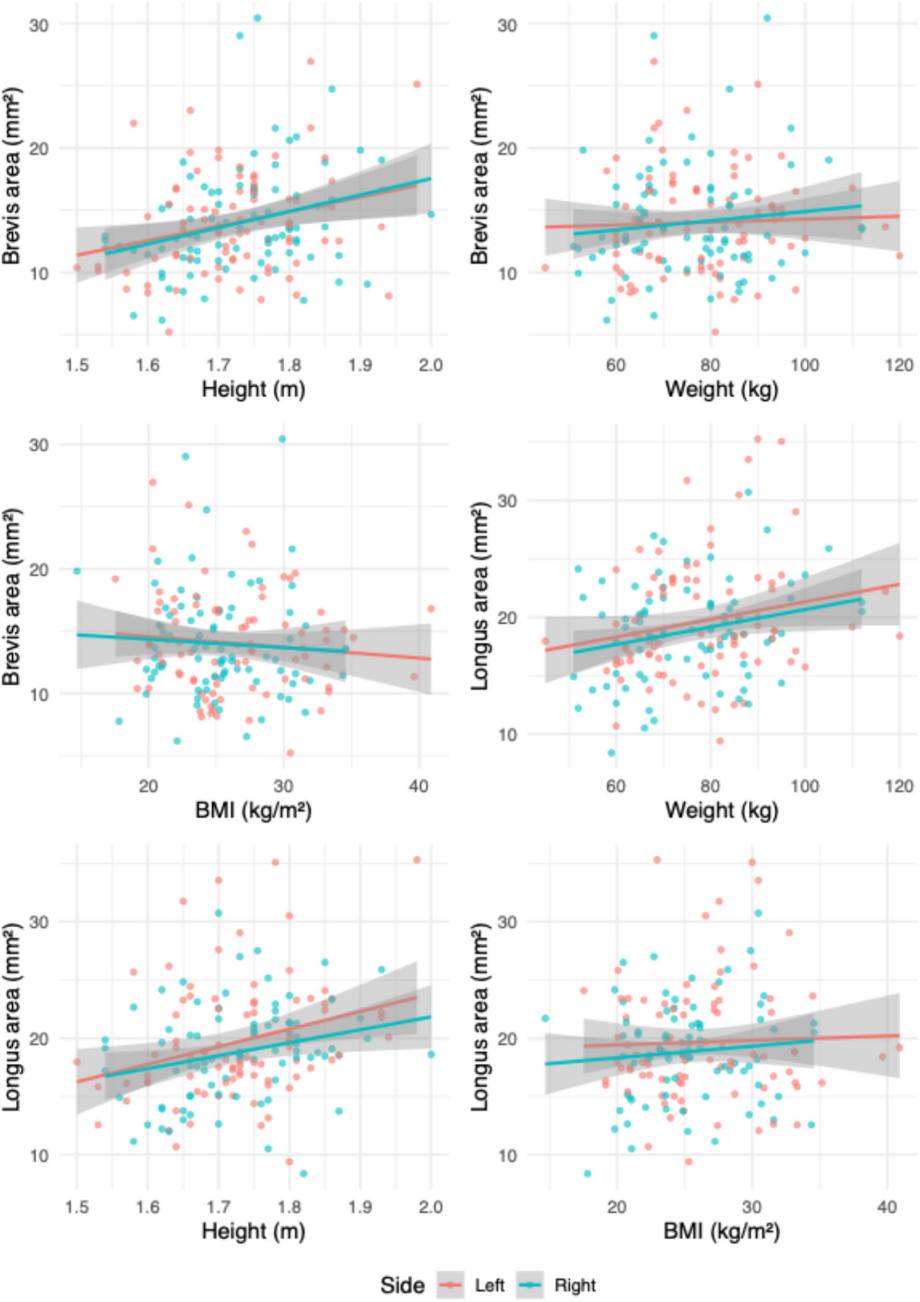
Association between anthropometric variables and peroneal tendon cross‐sectional area, stratified by side. Each panel shows a linear regression with the 95% confidence interval between tendon area (*y*‐axis) and height, weight, or body mass index (BMI) (*x*‐axis), separately for the peroneus brevis (top two rows) and peroneus longus (bottom row). Points are coloured by side (left/right). Regression lines indicate side‐specific trends and illustrate lateral consistency of the associations.

Multiple linear regression demonstrated significant associations between anthropometric variables and tendon cross‐sectional area. In the peroneus brevis model, both height (*β* = 12.9, *p* < 0.001) and age (*β* = 0.06, *p* < 0.001) were independently associated with a larger area. For the peroneus longus, only weight was a significant predictor (*β* = 0.07, *p* = 0.01); age and side were not. Models using BMI as the primary variable showed no significant associations. Regression coefficients with 95% CIs are reported in Table 2. Model fit and effect patterns are visualised in Figures 6, 7, 8, with additional unstratified and side‐stratified regressions shown in Figures 9 and 10.

**Table 2:** Linear regression models evaluating the association between anthropometric factors (height or weight), age, side and peroneal tendon cross‐sectional area.

Term	Estimate	Std.error	Statistic	*p* value	Conf low	Conf high	Model
Intercept	10.67	1.96	5.45	0	6.8	14.53	Brevis ~ Weight
Weight (kg)	0.01	0.02	0.49	0.62 (n.s.)	−0.04	0.06	Brevis ~ Weight
Age (years)	0.06	0.02	2.79	0.01	0.02	0.1	Brevis ~ Weight
Side—Right	0.02	0.65	0.03	0.98 (n.s.)	−1.27	1.3	Brevis ~ Weight
(Intercept)	−10.97	5.74	−1.91	0.06 (n.s.)	−22.3	0.35	Brevis ~ Height
Height (m)	12.93	3.26	3.97	0	6.5	19.36	Brevis ~ Height
Age	0.06	0.02	3.26	0	0.03	0.1	Brevis ~ Height
Side—Right	−0.07	0.62	−0.11	0.91 (n.s.)	−1.29	1.16	Brevis ~ Height
(Intercept)	13.78	2.19	6.29	0	9.45	18.1	Longus ~ Weight
Weight (kg)	0.07	0.03	2.8	0.01	0.02	0.13	Longus ~ Weight
Age	0	0.02	0.06	0.95 (n.s.)	−0.05	0.05	Longus ~ Weight
Side—Right	−0.65	0.73	−0.89	0.37 (n.s.)	−2.09	0.79	Longus ~ Weight
(Intercept)	−3.93	6.64	−0.59	0.55 (n.s.)	−17.03	9.18	Longus ~ Height
Height (m)	13.25	3.77	3.52	0	5.81	20.69	Longus ~ Height
Age	0.02	0.02	0.77	0.44 (n.s.)	−0.03	0.06	Longus ~ Height
Side—Right	−0.88	0.72	−1.22	0.22 (n.s.)	−2.29	0.54	Longus ~ Height

*Note*: Coefficients are unstandardised estimates (unstandardised regression coefficient) with standard errors and 95% confidence intervals.

Abbreviation: n.s., not significant.

### Sensitivity and robustness analysis

Robust regression using M‐estimation confirmed the stability of key associations. Effect estimates for height in the peroneus brevis model and weight in the peroneus longus model remained directionally and numerically consistent. Cook's distance plots indicated that no observation exceeded the 4/n threshold for influential points, although a few cases demonstrated moderate leverage. Excluding these observations and refitting the models yielded comparable results (Table 3). Diagnostic plots demonstrated acceptable linearity, homoscedasticity and approximate normality of residuals in both models, supporting the validity of the linear regression assumptions (Supporting Information S3: Figure A1).

**Table 3:** Sensitivity analyses using robust regression (M‐estimation with Huber loss) and refitting after exclusion of influential observations (Cook's distance > 4/n).

Term	Estimate	Std error	Statistic	Model	*p* value	Conf low	Conf high
(Intercept)	−9.7	5.39	−1.8	Robust Brevis	NA	NA	NA
Height (m)	12.24	3.06	4	Robust Brevis	NA	NA	NA
Age (years)	0.06	0.02	3.17	Robust Brevis	NA	NA	NA
Side—Right	−0.26	0.58	−0.45	Robust Brevis	NA	NA	NA
(Intercept)	14.52	2.12	6.84	Robust Longus	NA	NA	NA
Weight (kg)	0.06	0.03	2.25	Robust Longus	NA	NA	NA
Age	0	0.02	0.16	Robust Longus	NA	NA	NA
Side—Right	−0.3	0.71	−0.43	Robust Longus	NA	NA	NA
(Intercept)	−9.09	5.2	−1.75	Cleaned Brevis	0.08 (n.s.)	−19.36	1.19
Height (m)	12.06	2.97	4.06	Cleaned Brevis	0	6.2	17.93
Age	0.05	0.02	2.65	Cleaned Brevis	0.01	0.01	0.08
Side—Right	0.07	0.54	0.12	Cleaned Brevis	0.9 (n.s.)	−1	1.13
(Intercept)	13.84	2.09	6.61	Cleaned Longus	0	9.7	17.98
Weight (kg)	0.07	0.03	2.94	Cleaned Longus	0	0.02	0.12
Age	0	0.02	−0.12	Cleaned Longus	0.9 (n.s.)	−0.05	0.04
Side—Right	−0.37	0.7	−0.53	Cleaned Longus	0.59 (n.s.)	−1.75	1

*Note*: For robust models, *p*‐values and confidence intervals are not provided due to the estimation method. Results were consistent with standard models, supporting the stability of observed associations.

Abbreviation: n.s., not significant.

### Correlation between variables

**Figure 11:**
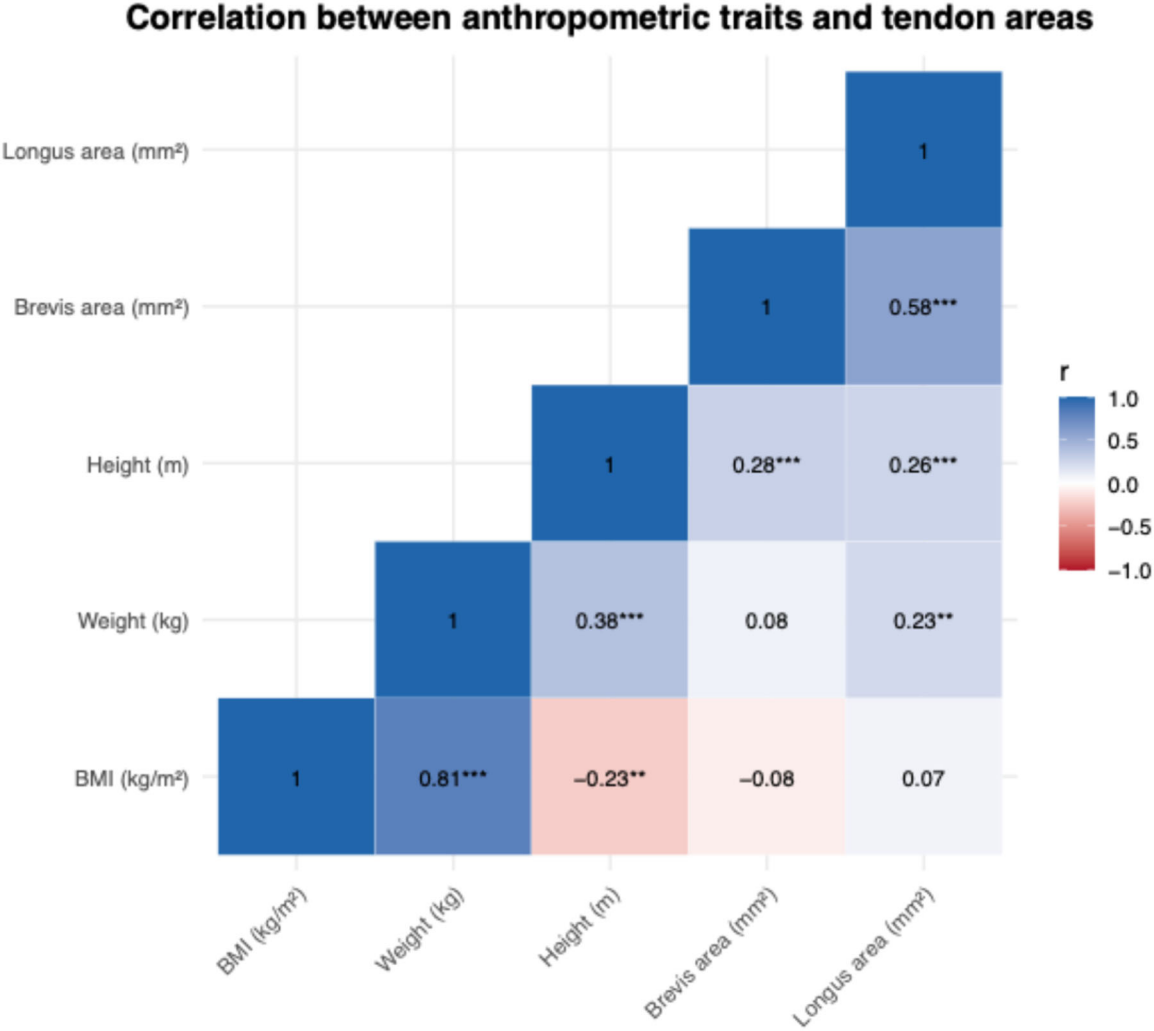
Correlation matrix showing Pearson coefficients between tendon areas and anthropometric variables. Pearson correlation coefficients are shown between body mass index (BMI), weight, height and cross‐sectional areas of the peroneus brevis and longus tendons. Asterisks indicate statistical significance: *p < 0.05, **p < 0.01; ***p < 0.001.

**Figure 12:**
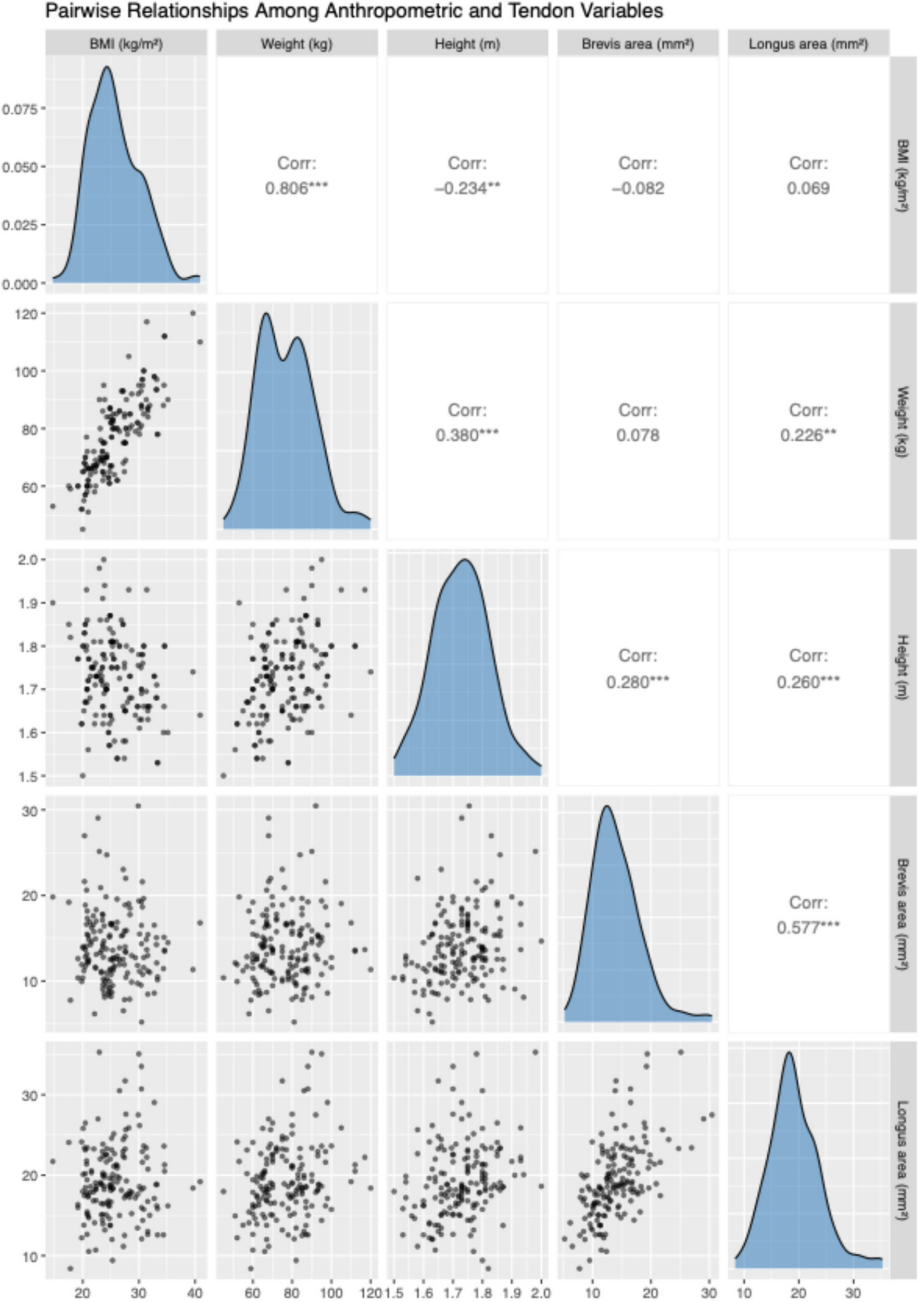
Scatterplot matrix showing all pairwise relationships among body mass index (BMI), weight, height and tendon areas. The lower triangle shows scatterplots, the diagonal shows density distributions and the upper triangle shows Pearson correlation coefficients.

Pearson correlation analysis revealed a strong positive correlation between the cross‐sectional areas of the peroneus brevis and longus tendons (*r* = 0.70, *p* < 0.001). Both tendon areas were moderately correlated with height (*r* = 0.48 for brevis, *r* = 0.41 for longus) and weight (*r* = 0.52 for brevis, *r* = 0.56 for longus). BMI showed weaker correlations with tendon area (*r* = 0.28–0.34). These relationships are visualised in the correlation matrix (Figure 11) and the full pairwise scatterplot matrix (Figure 12), demonstrating internal consistency and biological likelihood.

## WEIGHT‐BASED PREDICTION MODEL FOR PERONEUS LONGUS TENDON AREA

**Figure 13:**
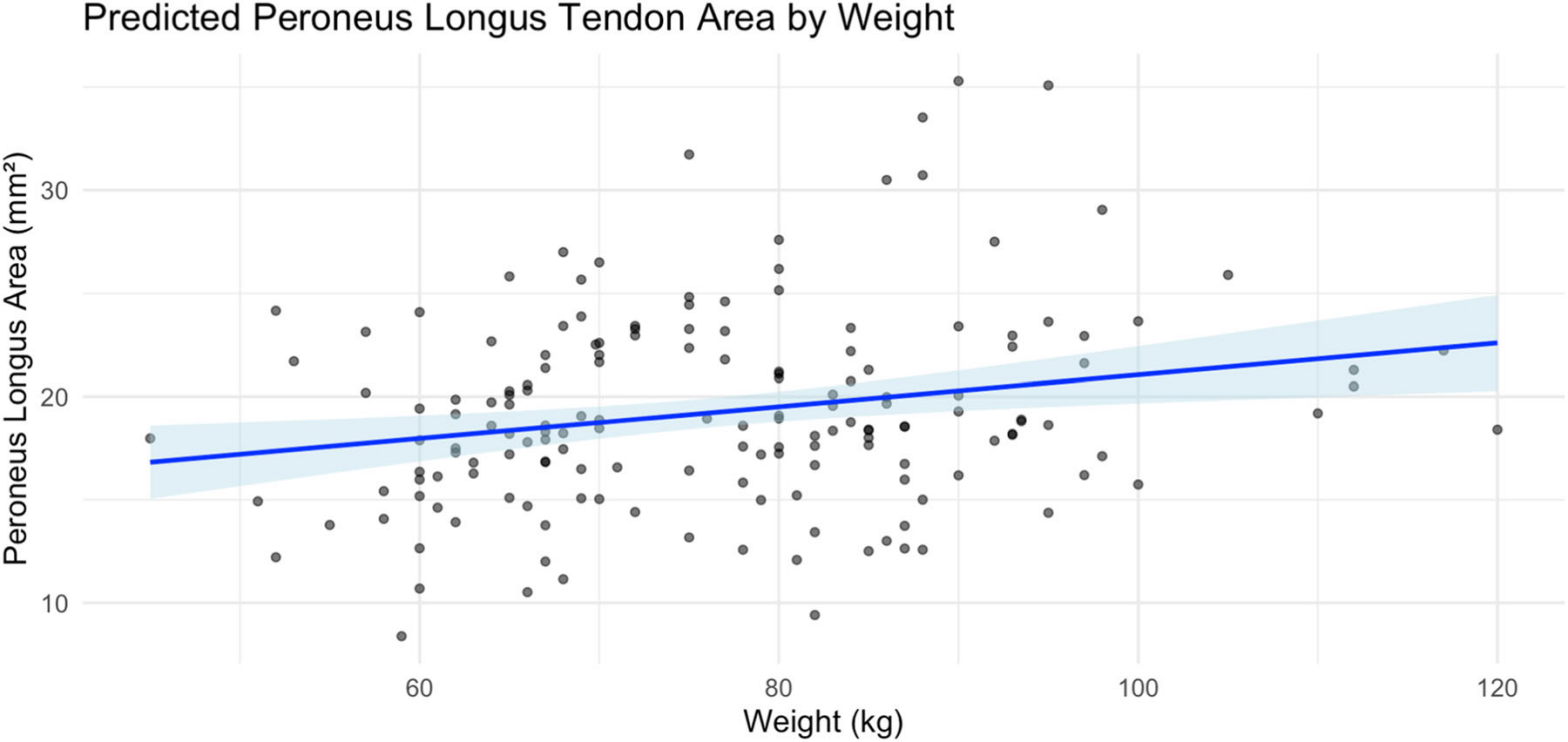
Linear regression model predicting peroneus longus tendon cross‐sectional area from weight. Each point represents an individual observation. The solid blue line indicates the fitted regression line, and the shaded area represents the 95% confidence interval. The model showed a statistically significant positive association between weight and tendon size (*p* = 0.003).

Univariable linear regression demonstrated a significant association between weight and the cross‐sectional area of the peroneus longus tendon (*β* = 0.077 mm²/kg, *p* = 0.003). The model intercept was 13.36 mm², yielding the following equation: Predicted area = 13.36 + 0.077 × weight (kg). The model explained 5.1% of the variance in tendon area (*R*² = 0.051). A table of predicted values across a range of body weights is provided in Table 4, and a regression plot is shown in Figure 13.

**Table 4:** Predicted cross‐sectional area of the peroneus longus tendon based on body weight.

Weight (kg)	Predicted peroneus longus cross sectional area (mm^2^)
50	17.20
55	17.59
60	17.97
65	18.36
70	18.74
75	19.13
80	19.51
85	19.90
90	20.28
95	20.67
100	21.05

*Note*: Estimates are derived from a linear regression model using weight (kg) as the sole predictor: Predicted cross‐sectional area of the peroneus longus tendon (mm²) = 13.36 + 0.077 × weight (kg).

## DISCUSSION

The most important finding of the present study was that height and weight are significantly associated with the cross‐sectional area of the peroneus brevis and longus tendons, as measured on MRI. To our knowledge, this is the first study to evaluate the cross‐sectional area of both tendons in a healthy adult population using a standardised, noninvasive imaging method. Previous studies were based on intraoperative measurements of graft diameter and focused exclusively on the peroneus longus tendon as a graft source for ACL reconstruction [5]. In contrast, our study utilised a large, nonsurgical cohort and standardised MRI‐based measurements with interrater reliability, offering a broader anatomical and clinical perspective. The analysis included both tendons: the peroneus longus due to its growing role in graft harvesting, and the peroneus brevis because of its critical function in ankle stability. Given that harvesting the peroneus longus may increase the mechanical load on the peroneus brevis, understanding the morphology of both tendons is essential for evaluating potential implications of tendon harvest on ankle biomechanics.

Peroneal tendon size is significantly associated with structural anthropometric variables of height and weight. In multivariable models, height emerged as an independent predictor of peroneus brevis cross‐sectional area, while weight was significantly associated with peroneus longus cross‐sectional area. These associations remained stable across sensitivity analyses and were consistent across sides, confirming the robustness of the anatomical relationships. All associations identified in the main regression models remained directionally consistent in robust regression and after excluding influential observations. Although widely used in clinical classification, BMI showed only weak and inconsistent associations with tendon size in analysed models. LOESS plots also did not reveal consistent nonlinear trends with tendon size. This pattern aligns with prior surgical studies investigating predictors of peroneus longus graft diameter. Rhatomy et al. reported a weak but statistically significant correlation between BMI and intraoperative graft diameter, suggesting a possible link between body composition and tendon size [19]. However, Wierer et al. found no independent association with BMI after multivariable adjustment and instead identified height as the most reliable predictor [25]. Similarly, Song et al. demonstrated that height and weight—rather than BMI—were significantly associated with peroneus longus graft diameter, and proposed a regression model for preoperative estimation [22]. These studies, despite methodological differences, converge on the conclusion that BMI alone is not a reliable anatomical predictor of tendon morphology.

The study results support the concept that tendon cross‐sectional area is more strongly associated with skeletal structure (height) than with adiposity (BMI), reflecting developmental and biomechanical adaptation. The weak or absent association between BMI and tendon area likely reflects differences in how various tendons respond to mechanical loading. In contrast to hamstring tendons, which often hypertrophy in individuals with higher BMI or greater muscle mass [25], peroneal tendons may reflect skeletal scaling rather than functional adaptation and appear less sensitive to overall body mass. The primary role of the peroneal tendons in lateral ankle stability and less direct involvement in large‐force generation could explain their more stable anatomical profile across BMI categories. The course of the peroneal tendons differs from that of the Achilles or hamstring tendons. Peroneal tendons glide over the lateral malleolus and are not subjected to vertical loading‐like tendons with a straight, axial trajectory. In obese patients, the Achilles tendon appears larger than it does in individuals with normal BMI [1]. This supports the need for tendon‐specific considerations when planning graft harvests and highlights the advantage of direct imaging over generalised anthropometric measures.

Preoperative estimation of the graft size helps to prevent graft failure [8]. When peroneal tendons are considered as autografts, as in ligament reconstruction, estimating their size based on BMI may lead to inaccurate assumptions. Our results suggest that height and weight are more reliable predictors of tendon dimensions and should guide graft suitability assessments. MRI‐based evaluation, when available, can provide individualised morphometric data, especially when traditional grafts such as hamstrings are not viable. Given that the peroneus brevis compensates for lost function after graft harvest, its size may influence long‐term outcomes and susceptibility to pathology [5, 18]. The susceptibility of the peroneus brevis to split tears may contribute to future symptoms or varus misalignment. The findings herein have implications not only for surgical planning but also for biomechanical modelling, risk assessment and training algorithm inputs in AI‐based imaging workflows.

Lastly, no significant difference in tendon size between left and right sides was observed, highlighting the bilateral anatomical symmetry of the peroneal tendons. This supports the validity of contralateral imaging as a reference in unilateral pathology and further attests to the consistency of our segmentation and analysis approach. The strong correlation between the peroneus brevis and longus cross‐sectional areas (*r* = 0.70) suggests coordinated development and shared mechanical scaling. The lack of side‐to‐side differences may be explained by the fact that, unlike the Achilles tendon, the peroneal tendons are not primary load‐bearing structures, and their morphology appears to be less influenced by limb dominance [3, 17].

This was a retrospective single‐centre study with several limitations. Anthropometric data were derived from self‐reported height and weight. While this could introduce bias, previous research has demonstrated high agreement between self‐reported and measured BMI in adults [7, 14]. Exclusion of individuals with tendinopathy, previous surgery, or imaging artefacts improved internal validity but may reduce generalisability to broader populations or pathologic states. Linear models were selected to balance interpretability and parsimony, although it is acknowledged that nonlinear or spline‐based approaches could be explored to further characterise subtle effects. Furthermore, tendon area does not directly reflect mechanical properties such as tensile strength or elasticity, which may differ based on microstructure and remains to be addressed.

We found that body weight is a significant predictor of peroneus longus tendon size and developed a simple formula to estimate tendon area based on weight alone. Although weight explains only a small portion of the variation, this model provides a quick and practical way to approximate graft size when MRI is unavailable. Height and BMI were less predictive in our cohort, suggesting that weight better captures relevant anatomical variation. This tool could assist surgeons in preoperative graft planning; however, tendon size is likely influenced by additional factors not captured in our model.

Whether the cross‐sectional area of the peroneus brevis after harvest is associated with biomechanical performance or risk of rupture remains to be determined. Moreover, it will be important to investigate how the extent of peroneus longus tendon harvesting affects ankle function and whether changes in the cross‐sectional area of the peroneus brevis after graft harvest correlate with biomechanical performance or risk of rupture [27]. Prospective studies combining mechanical testing with clinical outcomes would be valuable to clarify these relationships. Additionally, our dataset may provide a basis for developing automated tools to predict graft suitability based on MRI and anthropometric data, especially in clinical settings where real‐time intraoperative measurements are not feasible. Expanding this work to multicenter cohorts would further validate its generalisability across diverse populations.

## CONCLUSION

This study demonstrates that height and weight are significantly associated with the cross‐sectional area of the peroneus brevis and longus tendons on MRI. Greater height independently predicted a larger peroneus brevis area, while greater weight was associated with a larger peroneus longus area. In contrast, BMI—despite its widespread clinical use—was not an independent predictor of tendon size. These findings have direct implications for surgical planning: when considering harvesting a peroneus longus graft, reliance on BMI alone may be insufficient. Height and weight should be prioritised in the course of preoperative graft assessment. Furthermore, we provide a simple weight‐based formula to estimate peroneus longus tendon size, which may assist clinicians in preoperative graft planning when imaging is unavailable. Moreover, given that the peroneus brevis remains the primary dynamic stabiliser of the lateral ankle after peroneus longus harvest, its size may influence postoperative ankle function. MRI‐based tendon evaluation offers a noninvasive, anatomically precise tool to assess both graft suitability and residual stabilising capacity, supporting its use in individualised surgical planning.

## AUTHOR CONTRIBUTIONS

**Rafał Zych**: Methodology; software; data collection and data curation; writing—original draft; writing—review and editing. **Dan Mocanu**: Data Collection and data curation; writing—review and editing. **Katarzyna Bokwa‐Dąbrowska**: Methodology; data collection and data curation; writing—review and editing. **Dawid Dziedzic**: Methodology; data collection and data curation; writing—review and editing. **Pawel Szaro**: Conceptualisation; methodology; software; data collection and data curation; writing—original draft; writing – review and editing. All authors contributed to previous manuscript versions, and all have read and approved the final manuscript.

## CONFLICT OF INTEREST STATEMENT

The authors declare no conflicts of interest.

## ETHICS STATEMENT

All procedures performed in studies involving human participants were in accordance with the ethical standards of the institutional and/or national research committee and with the 1964 Helsinki Declaration and its later amendments or comparable ethical standards. The Swedish Ethical Authority approved this study and waived informed consent (number 2024‐07283‐02).

## Supporting information

Supporting material 1 macro.

Supporting Material 2 valid.

Supporting material 3 diagn.

## Data Availability

The data that support the findings of this study are available on request from the corresponding author. The data are not publicly available due to privacy or ethical restrictions.

## References

[1] Abate M . How obesity modifies tendons (implications for athletic activities). Muscles Ligaments Tendons J. 2014;4:298–302. 25489546 PMC4241419

[2] Albano D , Cortese MC , Duarte A , Messina C , Gitto S , Vicentin I , et al. Predictive role of ankle MRI for tendon graft choice and surgical reconstruction. Radiol Med (Torino). 2020;125:763–769. 32222954 10.1007/s11547-020-01177-z

[3] Benítez‐Martínez JC , Valera‐Garrido F , Martínez‐Ramírez P , Ríos‐Díaz J , Del Baño‐Aledo ME , Medina‐Mirapeix F . Lower limb dominance, morphology, and sonographic abnormalities of the patellar tendon in elite basketball players: a cross‐sectional study. J Athl Train. 2019;54:1280–1286. 31483151 10.4085/1062-6050-285-17 PMC6922568

[4] Bokwa‐Dabrowska K , Zych R , Mocanu D , Huuskonen M , Dziedzic D , Szaro P . Peroneus brevis split tear—A challenging diagnosis: a pictorial review of magnetic resonance and ultrasound imaging. Part 1. Anatomical basis and clinical insights. Eur J Radiol Open. 2025;14:100633. 39868415 10.1016/j.ejro.2024.100633 PMC11764704

[5] Butt U , Vuletic F , Shaikh MAA , Amanullah , Rehman G , Shah IA , et al. 5‐years outcomes following arthroscopic anterior cruciate ligament reconstruction comparing quadruple hamstring and peroneus longus tendon autografts: a randomized control trial. Arch Orthop Trauma Surg. 2024;145:85. 39714511 10.1007/s00402-024-05639-1 PMC11666688

[6] Davda K , Malhotra K , O'Donnell P , Singh D , Cullen N . Peroneal tendon disorders. EFORT Open Rev. 2017;2:281–292. 28736620 10.1302/2058-5241.2.160047 PMC5508858

[7] Davies A , Wellard‐Cole L , Rangan A , Allman‐Farinelli M . Validity of self‐reported weight and height for BMI classification: a cross‐sectional study among young adults. Nutrition. 2020;71:110622. 31837644 10.1016/j.nut.2019.110622

[8] Goyal S , Matias N , Pandey V , Acharya K . Are pre‐operative anthropometric parameters helpful in predicting length and thickness of quadrupled hamstring graft for ACL reconstruction in adults? A prospective study and literature review. Int Orthop. 2016;40:173–181. 26105766 10.1007/s00264-015-2818-3

[9] He J , Tang Q , Ernst S , Linde MA , Smolinski P , Wu S , et al. Peroneus longus tendon autograft has functional outcomes comparable to hamstring tendon autograft for anterior cruciate ligament reconstruction: a systematic review and meta‐analysis. Knee Surg Sports Traumatol Arthrosc. 2021;29:2869–2879. 32984919 10.1007/s00167-020-06279-9

[10] Imre N , Kocabiyik N , Sanal HT , Uysal M , Ozan H , Yazar F . The peroneus brevis tendon at its insertion site on fifth metatarsal bone. Foot Ankle Surg. 2016;22:41–45. 26869499 10.1016/j.fas.2015.04.009

[11] Jackson JB , Chu CH , Williams KA , Bornemann PH . Normal ultrasonographic parameters of the posterior tibial, peroneal, and achilles tendons. Foot & Ankle Specialist. 2019;12:480–485. 30264576 10.1177/1938640018800785

[12] Kim HN , Jeon JY , Dong Q , Noh KC , Chung KJ , Kim HK , et al. Lateral ankle ligament reconstruction using the anterior half of the peroneus longus tendon. Knee Surg Sports Traumatol Arthrosc. 2015;23:1877–1885. 24841944 10.1007/s00167-014-3072-8

[13] Koo TK , Li MY . A guideline of selecting and reporting intraclass correlation coefficients for reliability research. J Chiropr Med. 2016;15:155–163. 27330520 10.1016/j.jcm.2016.02.012 PMC4913118

[14] Lassale C , Péneau S , Touvier M , Julia C , Galan P , Hercberg S , et al. Validity of web‐based self‐reported weight and height: results of the Nutrinet‐Santé study. J Med Internet Res. 2013;15:e152. 23928492 10.2196/jmir.2575 PMC3742400

[15] Mocanu D , Bokwa‐Dąbrowska K , Nilsson Helander K , Szaro P . Comparative analysis of ultrasound and magnetic resonance imaging in diagnosing pain in the posterolateral region of the ankle. J Ultrason. 2025;25(100). 10.15557/jou.2025.0002 PMC11893017 40066264

[16] Patil V , Frisch NC , Ebraheim NA . Anatomical variations in the insertion of the peroneus (fibularis) longus tendon. Foot Ankle Int. 2007;28:1179–1182. 18021587 10.3113/FAI.2007.1179

[17] Petrovic I , Amiridis IG , Holobar A , Trypidakis G , Kellis E , Enoka RM . Leg dominance does not influence maximal force, force steadiness, or motor unit discharge characteristics. Med Sci Sports Exerc. 2022;54:1278–1287. 35324535 10.1249/MSS.0000000000002921

[18] Rhatomy S , Asikin AIZ , Wardani AE , Rukmoyo T , Lumban‐Gaol I , Budhiparama NC . Peroneus longus autograft can be recommended as a superior graft to hamstring tendon in single‐bundle ACL reconstruction. Knee Surg Sports Traumatol Arthrosc. 2019;27:3552–3559. 30877316 10.1007/s00167-019-05455-w

[19] Rhatomy S , Tanzil H , Setyawan R , Amanda C , Phatama KY , Andrianus J , et al. Influence of anthropometric features on peroneus longus graft diameter in anterior cruciate ligament reconstruction: a cohort study. Ann Med Surg. 2019;48:77–80. 10.1016/j.amsu.2019.10.023 PMC6849122 31737263

[20] Rhatomy S , Wicaksono FH , Soekarno NR , Setyawan R , Primasara S , Budhiparama NC . Eversion and first ray plantarflexion muscle strength in anterior cruciate ligament reconstruction using a peroneus longus tendon graft. Orthop J Sports Med. 2019;7:2325967119872462. 31632995 10.1177/2325967119872462 PMC6767728

[21] Shao X , Shi LL , Bluman EM , Wang S , Xu X , Chen X , et al. Satisfactory functional and MRI outcomes at the foot and ankle following harvesting of full thickness peroneus longus tendon graft. Bone Jt J. 2020;102–B:205–211. 10.1302/0301-620X.102B2.BJJ-2019-0949.R1 32009424

[22] Song X , Li Q , Wu Z , Xu Q , Chen D , Jiang Q . Predicting the graft diameter of the peroneus longus tendon for anterior cruciate ligament reconstruction. Medicine. 2018;97:e12672. 30383628 10.1097/MD.0000000000012672 PMC6221677

[23] von Elm E , Altman DG , Egger M , Pocock SJ , Gøtzsche PC , Vandenbroucke JP . The strengthening the reporting of observational studies in epidemiology (STROBE) statement: guidelines for reporting observational studies. Lancet. 2007;370:1453–1457. 18064739 10.1016/S0140-6736(07)61602-X

[24] Wang XT , Rosenberg ZS , Mechlin MB , Schweitzer ME . Normal variants and diseases of the peroneal tendons and superior peroneal retinaculum: MR imaging features. Radiographics. 2005;25:587–602. 15888611 10.1148/rg.253045123

[25] Wierer G , Gwinner C , Scheffler S . Peroneus longus split versus semitendinosus tendon autograft size: a cross‐sectional study. Am J Sports Med. 2023;51:1743–1751. 37092720 10.1177/03635465231165297

[26] Zych R , Dziedzic D , Bokwa‐Dąbrowska K , Mocanu D , Szaro P . MRI evaluation of peroneus brevis tendon position: anatomical variants in individuals with normal peroneal tendons to improve recognition and prevent misdiagnosis. Ann Anat Anatom Anzeiger. 2025;261:152694. 10.1016/j.aanat.2025.152694 40651667

[27] Zych R , Mocanu D , Hagberg Y , Bokwa‐Dąbrowska K , Dziedzic D , Helander KN , et al. Association between flat variants of the peroneus brevis tendon and split tears on magnetic resonance imaging. Skelet Radiol. 2025. 10.1007/s00256-025-05032-y PMC12743021 40940445

[28] Zych R , Mocanu D , Hagberg Y , Bokwa‐Dąbrowska K , Huuskonen M , Romanus I , et al. Validation of an MRI‐based classification of peroneus brevis tendon morphology: a four‐type system with high inter‐rater reliability. Skelet Radiol. 2025. 10.1007/s00256-025-05010-4 PMC12627172 40796683

