# Supplementary material for: Height and weight predict cross‐sectional area of the peroneus brevis and longus tendons: Magnetic resonance imaging‐based analysis of 164 adults
Source: J Exp Orthop. 2025 Nov 3;12(4):e70492. doi: 10.1002/jeo2.70492 (PMC12581844; doi:10.1002/jeo2.70492)
Supplement: Supplementary file 2 — Supporting Material 2 valid. [file JEO2-12-e70492-s001.docx]

# Supplementary material 2

## Macro validation

To validate the reliability of the custom ImageJ macro used for tendon segmentation, a board-certified musculoskeletal radiologist re-segmented 60 images after a four-week interval. Intra-rater agreement was excellent, with an intraclass correlation coefficient (ICC[A,1]) of 0.996 (95% CI: 0.993–0.998). Bland–Altman analysis showed a mean difference of –0.15 mm² with limits of agreement from –1.05 to 0.76 mm². The Pearson correlation coefficient was 0.996 (95% CI: 0.994–0.998, p < 0.001). The median absolute difference between repeated measurements was 0.35 mm² (range: 0.008–1.26 mm²).
