# Supplementary material for: Height and weight predict cross‐sectional area of the peroneus brevis and longus tendons: Magnetic resonance imaging‐based analysis of 164 adults
Source: J Exp Orthop. 2025 Nov 3;12(4):e70492. doi: 10.1002/jeo2.70492 (PMC12581844; doi:10.1002/jeo2.70492)
Supplement: Supplementary file 3 — Supporting material 3 diagn. [file JEO2-12-e70492-s002.docx]

# Supplementary material 2

### Model diagnostics

Visual inspection of regression diagnostics for the final peroneus brevis (height model) and peroneus longus weight model indicated acceptable model fit. Residuals were approximately symmetrically distributed with no evidence of major skewness or heteroscedasticity. Q–Q plots suggested mild deviation from perfect normality, particularly in the upper tails, but the patterns remained within acceptable bounds. Cook’s distance identified a few high-leverage observations (e.g., IDs 165, 164, 151), yet none exceeded conventional influence thresholds (4/n), supporting model stability (Supplementary Figure A1).

**Supplementary Figure A1.** Regression diagnostics for the final peroneus brevis (height model) and peroneus longus weight model, including residuals vs fitted, Q–Q plot, scale-location plot, and Cook’s distance. Residuals showed mild deviation from normality without major outliers.
